# Reliability and Validity of the Persian Version of Templer Death Anxiety Scale-Extended in Veterans of Iran–Iraq Warfare

**Published:** 2014

**Authors:** Hamid Sharif Nia, Abbas Ebadi, Rebecca H Lehto, Batool Mousavi, Hamid Peyrovi, Yiong Huak Chan

**Affiliations:** 1 Behavioral Sciences Research Center, School of Nursing, Baqiyatallah University of Medical Sciences AND Janbazan Medical and Engineering Research Center (JMERC), Tehran, Iran.; 2 Associate Professor, Behavioral Sciences Research Center AND Department of Nursing, School of Nursing, Baqiyatallah University of Medical Sciences AND Janbazan Medical and Engineering Research Center (JMERC), Tehran, Iran.; 3 ssistant Professor, Michigan State University, School of Nursing, East Lansing, MI, USA.; 4 Specialist, Department of Prevention, Janbazan Medical and Engineering Research Center, Tehran, Iran.; 5 Associate Professor, Center for Nursing Care Research, Department of Critical Care Nursing, School of Nursing and Midwifery, Iran University of Medical Sciences, Tehran, Iran.; 6 Head, Biostatistics Unit, Yong Loo Lin School of Medicine, National University Health System, Singapore

**Keywords:** Death Anxiety, Reliability, Templer Death Anxiety Scale, Validity, Veteran

## Abstract

**Objective:**

The purpose of this study was to determine the validity and reliability of the Persian version of Templer Death Anxiety Scale-Extended (DAS-E) in veterans of Iran**–**Iraq Warfare.

**Methods:**

In this cross-sectional study, 211 male veterans of Iran**–**Iraq Warfare completed the 51 item DAS-E. Principal components analysis with varimax rotation was used to assess domain structure of the DAS-E. Internal consistency reliability was assessed with Cronbach’s alpha. Test–retest reliability was assessed with intra-class correlation coefficients for absolute agreement for the individual items and domains.

**Results:**

The construct validity of the scale was obtained using exploratory factor analysis that showed four factors with Eigen values of greater than 1 (1, 11 items, α = 0.83; 2, 7 items; α = 0.87; 3, 5 items, α = 0.73; and 4, 4 items, α = 0.75). Test–retest and internal consistency (total alpha) was 0.91 and 0.89, respectively.

**Conclusion:**

The DAS-E demonstrated suitable validity and reliability among the veterans under study. The factor analysis demonstrated that the DAS-E has a multi-dimensional structure. With consideration of the proper psychometric characteristics, this scale can be used to further research about death anxiety in this population.

## Introduction

Fear of death is commonly experienced by humans (1) and is a major human concern that has motivated both the humanities and psychological inquiry over the past centuries (2). Due to increasing recognition of the importance of death anxiety toward understanding human behavior, death anxiety has evolved into an important branch of thanatology research (3). Humans, unlike other creatures, are aware of personal mortality and this knowledge can cause fear of death and dying leading to the anxiety provocation (4). The term, death anxiety, refers to the anxiety about death experienced in “daily life” and not to the anxiety that is felt in the case of an urgent risk towards a person’s life (5). One common definition of death anxiety is expressed as a negative emotional reaction provoked by the anticipation of a state in which the self does not exist (6). In the health care environment, death anxiety is “an important concept to consider in a wide range of practice settings, including community cancer screenings of healthy individuals, psychiatric care, acute and trauma care, chronic care, and pediatrics and in individuals facing diagnosis of a life-threatening illness” (2).

Because of its importance to human responses to health stressors, death anxiety is included as a nursing diagnosis with specific North American Nursing Diagnosis Association nursing outcome criteria (2). It is essential that researchers who investigate death anxiety have reliable and valid methodology to measure the phenomenon in their population of interest (7). Iran**–**Iraq war veterans who sustained trauma and injuries in the battlefield are a vulnerable population with a myriad of physical and psychological co-morbidities. Despite this awareness, little research has been conducted better to understand the impact of death anxiety on the veterans' current function. One reason for the limited research is that there have not been validated death anxiety tools to capture this phenomenon in this population (7, 8).

Many human factors have been associated with death anxiety. In a meta-analysis of death anxiety research, it was shown that age, ego integrity, institutionalization, physical and mental health disorders, and religion were associated with the phenomena (9). Another study demonstrated that ethnicity, occupational exposures to death scenes, and personal experiences associated with death all have a connection to the level of death anxiety (10). Given this impact, it has been increasingly recognized that is an essential responsibility across cultures to assist patients and their family members to adapt to the reality of death and to manage associated behavioral and emotional consequences (11).

Human attitudes toward death are expressed either consciously or unconsciously by personal characteristics and via culture, social and philosophical belief systems (12). Religious institutions carry rituals, theological and philosophical views, and mythologies that are often culturally determined. These cultural perspectives play a strong role in the development of different death attitudes among individuals who belong to the various religious orientations (13). In addition, the meaning of death may vary within the same culture, because of individual differences in personality characteristics, traits, and beliefs that shape a person’s conscious or unconscious behaviors and attitudes to death and dying (1, 12). Researchers have identified that death anxiety is a multidimensional construct (14). Dimensions of death anxiety include fear of decay and body decline, adolescent or premature death, and fears of being falsely declared dead (15). Death anxiety occurs when the dimensions are activated in situations that include uncertainty, fear of pain, isolation, corpse anxiety, anxiety about life after death, and related to unfinished business (16, 17). Death anxiety influences both personality and psychological health and may contribute to existential crises (7). However, if individuals have positive perceptions about their situation in life they may have reduced death anxiety (18).

Appropriate methodology is critical to the study of death anxiety. Because death anxiety is often measured by self-report and there are multiple instruments available to test the construct (19) it is essential that standardized instruments with strong psychometrics are used (7). Death anxiety questionnaires can be classified into uni-dimensional and multi-dimensional scales (20). The uni-dimensional Death Anxiety Scale (DAS) (21) prepared by Templer has been most commonly used in death anxiety research (2). Although the DAS has been used in approximately 60% of the studies on the topic (21), it has not been validated in an Iranian population. The DAS construction and validation was completed in 1967, presented in 1969, and published in 1970. A later version, the DAS-extended, was developed in 2006. The 51-item DAS-E was generated to extend the content validity of the DAS, early testing has demonstrated correlation at the 0.001 level in three out of four groups of participants (one Kuwaiti, one Sudanese, and two American) (22).

On 22^nd^ September 1980, the Iraqi army attacked Iran, and the ensuing military conflict continued until August 1988. The Iraq-Iran war was one of the greatest conflicts in the 20^th^ century, resulting in scores of deaths and injuries to many military and non-military affiliated people (23). Examining the veteran population and their fear of death is deemed necessary as veterans are an underrepresented population of interest in the literature on death and dying (10). Salamati et al. reported that 217,489 to 188,015 Iranians were martyred during the war, and by the end of the war, 398,587 people were injured. Among the injured were 52,000 chemical warfare victims (24). Despite the passage of 26 years since the cease-fire, the mental and physical health disabilities that are experienced by surviving war veterans remain a significant health care system challenge (8). Thus, the aim of this study is to present validity and reliability findings of the Persian version of the 51-question extended version of the DAS among Iranian Iran**–**Iraq war veterans many of whom were battle disabled (chemical weapons exposed, physical disability) victims.

## Materials and Methods

***Development of the questionnaire content***

This cross-sectional study included a total sample of 211 male Iran**–**Iraq war veterans. Although standardized rules regarding sample size for exploratory factor analysis (EFA) have not yet been established, a common standard is an attainment of a sample size of at least 100 participants (25). A ratio of at least 10 subjects for each factor is desirable to generalize from the sample under study to a wider population. Thus, the sample size yielding 100-200 subjects is adequate for the proposed factor analysis (26). Inclusion criteria included adult men who had participated in Iran**–**Iraq war between the years 1980 and 1988 who have war-related disabilities. Exclusion criteria include male veterans who did not participate in the Iran–Iraq war; and veterans with clinically validated psychiatric disorders, for example, post-traumatic stress disorder that would negatively impact their ability to participate in the proposed research. Data were collected from August 2012 to January 2013 after receiving human studies Ethical Committee approval from the Baqiyatallah University of Medical Sciences. All participants provided informed consent after the study purpose was explained, and assurances were made of anonymity and privacy. Prior to its implementation, the DAS-E was first translated into the Persian (Farsi) language, and English professors, psychologists and psychiatrists tested the survey. The former DAS is available in two formats: true-false or Likert scale format (27). In the current study, the DAS-E consists of 5-items, which are based on a Likert-scale format (scale of 1-5, strongly disagree to strongly agree). Five DAS-E questions are scored inversely (items 2, 3, 6, 7, and 15). Thus, the total score ranges between 51 and 255 items. A higher score indicates higher levels of death anxiety.

***Statistical analysis***

*Equivalence and stability reliability*

In order to assess the reliability, the internal consistency was calculated using Cronbach’s (α). Instruments with Cronbach’s α value of 0.70 or greater are considered to have satisfactory internal consistency (28). Some authors have recommended that the coefficient alpha should be minimally 0.90, with an ideal value of 0.95 (29, 30). Other experts have contended that alpha coefficient values over 0.90 reflect redundancies and indicate that the tool should be shortened (31). Internal consistency indicates how well the items on an instrument fit together conceptually (32). To estimate the suitable sample size for the test–retest reliability, power analysis was performed. The power analysis identified that a sample of 15 veterans was required to have power of 0.80 to detect a test–retest correlation of 0.90 at a significance level of 0.05 (33, 34).

To examine the test–retest reliability of the DAS-E, a sub-sample of veterans (n = 15) completed the questionnaire twice between a 2-week interval. The test–retest reliability of the recall ratings was assessed using two-way mixed intra-class correlation coefficients (ICC) for absolute agreement at the level of individual items where the ICC of 0.4 or more was considered as acceptable (35). For the test–retest reliability, 2 weeks to 1 month is the generally accepted time interval for retesting (32).

*Construct validity*

To examine the construct validity, the performance of the questionnaire was tested using EFA. Any missing values were replaced with the mean. The study sample consisted of 211 veterans who completed the questionnaire. The instrument’s factor structure was extracted using principal component analysis (PCA) with varimax rotation. The Kaiser–Meyer–Olkin (KMO) and Bartlett’s test of sphericity were used to examine the appropriateness of the sample for the factor analysis. The number of factors extracted was determined and evaluated using the scree plot (36). Factor loadings equal or greater than 0.5 were considered as appropriate (37).

Kaiser recommended accepting values >0.5 and described values between 0.5 and 0.7 as mediocre; 0.7 and 0.8 as good, 0.8 and 0.9 as great, and >0.9 as superb (38). Furthermore, Steven suggested that a factor is reliable if it has 10 or more variables with loadings of 0.4 and ≥150 participants (39).

P value < 0.05 were considered as statistically significant. The analyses were performed using SPSS for Windows (version 16.0 SPSS Inc., Chicago, IL, USA).

## Results

The mean age of the 211 male veterans was 48.99 (SD = 4.62) years. Of this sample, 69 men (32.70%) had physical disabilities, 71 men (33.60%) experienced chemical weapon exposure, and 71 men (33.6%) had both physical disabilities and a history of chemical weapon exposure. The mean disabilities was 34.60% (SD = 15.80), range 10-70% disabilities. Among these veterans, most were married (93.80%, n = 198), and most had a diploma level of education (30.30%, n = 64) equivalent to a high school (12 years) level in other countries.

***Reliability***

The instrument had a high internal consistency (Cronbach’s α = 0.89). The ICC was 0.91 (95% CI: 0.86-0.95, p < 0.001) indicating appropriate stability for the questionnaire.

***EFA***

**Figure 1:**
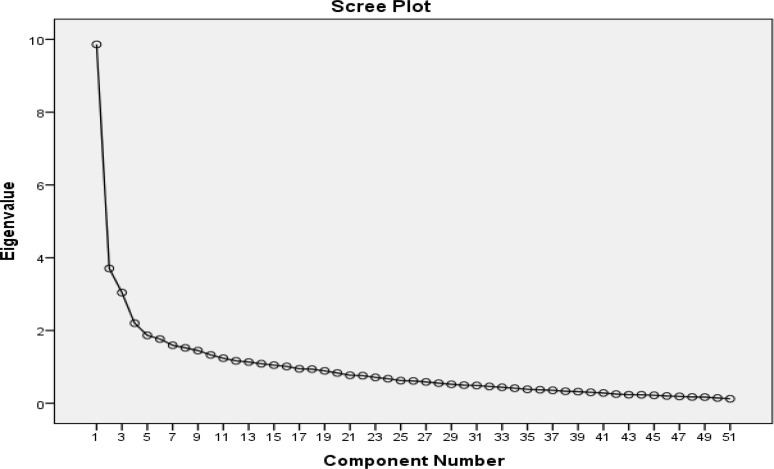
Scree plot by principle component analysis of the Death Anxiety Scale-Extended (n = 211)

The KMO was 0.78, and the Bartlett’s test of sphericity was significant (4535.07, p < 0.001) demonstrating that the sampling was adequate. Principal factor analysis with varimax rotation was conducted to assess the underlying structure for the 51-items of the DAS-E. The scree plot (Figure 1) indicated that a four-factor solution was optimal. The initial analysis (before varimax rotation) indicated a six factors structure for the questionnaire. After varimax rotation, eleven items were loaded on the first factor, which explained 16.5% of the rotation variance. Seven items were loaded on the second factor with 9.5% of rotation variance. Five items were loaded on the third factor with 7.9% of rotation variance. Four items were loaded on the fourth with 6.7% of rotation variance. The total cumulative variance explained by these four factors was 40.6%. Table 1 displays the items and factor loadings for the rotated factors, with loadings less than 0.50 omitted to improve clarity.

**Table 1:** Exploratory factor loadings of items in the Templer Death Anxiety Scale-Extended with four factors

**Factor**	**Item**		**h** ^2^ †	**% of variance**	**Eigen values** ‡	**Cronbach’s α** *
**First**	I often dream about death		0.870	16.50	9.35	0.83
	I would not let doctors treat me because sometimes they accidentally kill people		0.758			
	When I am in small places I worry about being trapped and dying		0.754			
	It makes me nervous when I see an ambulance		0.742			
	I am afraid of being killed in my sleep		0.634			
	I am afraid of sleeping alone		0.622			
	I avoid stories involving death		0.613			
	I have nightmares about dying. Dreams about dying often wake me		0.611			
	When I think about death I cannot go to sleep		0.598			
	I am very much afraid of a terrorist attack		0.540			
	I do not like being around people who are very old		0.537			
	Worried aggravated to death(or pessimistic worry)					
**Second**	I am really scared of having a heart attack		0.686	9.50	4.82	0.87
	I am afraid of dying on a hi-jacked plane		0.653			
	I am afraid of dying in an accident		0.606			
	I am afraid of dying from a life-threatening disease		0.593			
	I fear drowning		0.582			
	I am afraid of being embalmed		0.511			
	I fear dying a painful death		0.504			
	Fear of destruction					
**Third**	I very much fear being tortured to death		0.699	7.90	4.02	0.73
	Dreams that bother me involve death		0.659			
	I worry that I might die today		0.641			
	I am afraid of being burned or cremated while I am still alive		0.550			
	I very much fear burning in hell		0.537			
	Agony of death					
**Fourth**	I am not at all afraid to die	-0.726		6.70	3.37	0.75
	I am very much afraid to die		0.634			
	The thought of death never bothers me	-0.584				
	Movies involving people dying trouble me		0.568			
	Sense of ending					

† Extraction (final) communalities (row sums of squared loading);

‡ Rotation column sums of squared loadings

* Reported for per factor after varimax rotations

## Discussion

The purpose of the study was first to translate and then conduct reliability and validity testing of the DAS-E in Iran**–**Iraq war veterans, all of whom who had chemical exposures or war disabilities. The findings demonstrated that the DAS-E is a multidimensional instrument.

The study indicated that the instrument’s overall reliability approximated excellence. Importantly, the alpha Cronbach coefficients were calculated for the four factors separately. High values for Cronbach’s alpha indicated good internal consistency of the items in the four factors. Higher Cronbach’s alpha indicated a high correlation between the items and showed that the questionnaire is consistently reliable (40).

Importantly increasing the value of alpha is partially dependent upon the number of items in the instrument. Although a high value for Cronbach’s alpha does not mean that the scale is uni-dimensional, Templer reported 0.83 coefficients for a test–retest reliability and 0.76 for internal consistency coefficients (Kuder–Richardson formula 20) (41). Kelly and Corriveau reported 0.85 test–retest and 0.73 internal consistency coefficients (42). Also, Abdel-Khalek reported 0.57 split-half reliability coefficients for males and 0.78 for females in an Arabic version of DAS (43). Furthermore, Al-Arja and Abdallah found 0.92 Cronbach’s alpha coefficients in their research (44). In the study by Saggino and Kline, who had calculated 0.75 Cronbach’s alpha coefficients for the total score, three factors commutated less high (α < 0.7) (45).

Using PCA and varimax rotation, factor analysis results demonstrated that four factors of Eigen values were higher than one. In addition, these four factors expressed 40.6% of the total variance, with the first factor (16.5%) carrying the highest share. Therefore, the DAS-E is not a uni-dimensional scale for this population, and may include several factors, that have been affirmed by other researchers (46). Thorson and Powell found four factors: 1) fear of isolation and immobility (51.7% of the variance); 2) fear of pain (11.8%); 3) fear of the finality of death (16.5%); and 4) fear of burial and decomposition (12.7%) (47). Abdel-Khalek et al. (48), and Levin (49) in their research obtained five factors, Abdel-Khalek found four factors (50), and Saggino and Kline reported three factors for DAS and introduced it as a multidimensional scale (45). Thorson and Perkins had indicated four principal factors: 1) fear of isolation and immobility; 2) fear of pain; 3) fear of the finality of death, and 4) fear of burial and decomposition (51). Tavakoli and Ahmadzadeh also introduced five factors (first factor: absolute death anxiety; second factor: fear of patience and pain; third factor: death related thoughts; fourth factor: time passing and short life; and fifth factor: fear of future) in the analysis of DAS, which indicated 51.4% common variance (7). Thakur and Thakur found that some of the items of Templer’s DAS such as, “I am often distressed by the way time flies so very rapidly," were not fear generating for Indian respondents. They commented that this item possibly applies better in economic security than for death anxiety (52). In addition, it is essential to note that there are specific items that may provoke death anxiety in predominantly Muslim societies. For instance, Abdel-Khalek distinguished that the “torture of the grave” was among the highest mean score item relative to the fear of death. This item, however, is not well known outside the belief of Islam (53).

In the Islamic faith, there is a belief in the afterlife. The afterlife is an eternal and immortal life. Afterlife is imminent and begins in the grave directly after burial (21). By and large, a person‘s thinking and behavior may be influenced more than we can distinguish by views, hopes and anxiety concerning the nature and meaning of death (54). Nevertheless, Pollak concludes that: “Death anxiety is a complex construct that interrelates in a variety of ways that are not completely understood with a host of demographic and personality variables” (p. 119) (55). Further, death anxiety, a human specific characteristic, is likely formed in tandem with higher-level cognitive structures, thus impacting prediction and prophecy for human beings (11, 56).

This study has some advantages and limitations. Inclusion of groups of experts (translators, statistician expert, and methodologists) in the study design and a strong assessment of the questionnaire is a strong point. This study has several limitations. For example, convenience sampling, selection bias. Another limitation resides in that the DAS-E relies on self-report and thus does not capture implicit processes. Importantly, conducting rigorous reliability and validity testing can offset problems associated with poorly designed questionnaires (57). 

## Conclusion

The results indicate that the DAS-E has a solid reliability and validity in Persian edition, and demonstrates a multidimensional structure. Importantly, future research should incorporate continued testing of the instrument in other Iranian populations. In recent years, death anxiety has been increasingly considered as a multidimensional construct (58). The terror management theory suggests that self-esteem and cultural worldviews help buffer the effects of death anxiety, by this means lessening the anxiety that develops when one is reminded that life is finite (59). Having a valid and reliable tool is an important step in carrying out studies that can be used to understand death anxiety in affected Iranian individuals, in particular Iranian war veterans directly impacted by traumatic exposures, so as to better understand how best to develop interventions to help individuals from this culture.

## Notes

**Declaration of interest:** None.
